# Pregnancy in arrhythmogenic cardiomyopathy

**DOI:** 10.1007/s00399-021-00770-7

**Published:** 2021-05-25

**Authors:** Thomas Wichter, Peter Milberg, Henry D. Wichter, Dirk G. Dechering

**Affiliations:** Klinik für Innere Medizin / Kardiologie, Niels-Stensen-Kliniken, Marienhospital Osnabrück, Herzzentrum Osnabrück/Bad Rothenfelde, Bischofsstr. 1, 49074 Osnabrück, Germany

**Keywords:** ARVC, Maternal outcomes, Right ventricle, Genetic counseling, Ventricular tachycardia, ARVC, Maternales Outcome, Rechter Ventrikel, Genetische Beratung, Ventrikuläre Tachykardie

## Abstract

Arrhythmogenic cardiomyopathy (AC) is a rare heart muscle disease with a genetic background and autosomal dominant mode of transmission. The clinical manifestation is characterized by ventricular arrhythmias (VA), heart failure (HF) and the risk of sudden cardiac death (SCD). Pregnancy in young female patients with AC represents a challenging condition for the life and family planning of young affected women. In addition to genetic mechanisms that influence the complex pathophysiology of AC, experimental and clinical data have confirmed the pathogenetic role of strenuous exercise and competitive sports in the early onset and rapid progression of AC symptoms and complications. Pregnancy and exercise share a number of physiological aspects of adaptation. In AC, both result in ventricular volume overload and myocardial stretch. Therefore, pregnancy has been postulated as a potential risk factor for HF, VA, SCD, and pregnancy-related obstetric complications in patients with AC. However, the available evidence on pregnancy in AC does not confirm this hypothesis. In most women with AC, pregnancies are well tolerated, uneventful, and follow a benign course. Pregnancy-related symptoms (VA, syncope, HF) and mortality, as well as obstetric complications, are uncommon in AC patients and range in the order of background populations and cohorts with AC and no pregnancy. The number of completed pregnancies is not associated with an acceleration of AC pathology or an increased risk of VA or HF during pregnancy and follow-up. Accordingly, there is no medical indication to advise against pregnancy in patients with AC. Preconditions include stability of rhythm and hemodynamics at baseline, as well as clinical follow-ups and the availability of multidisciplinary expert consultation during pregnancy and postpartum. Genetic counseling is recommended prior to pregnancy for all couples and their families affected by AC.

**Supplementary Information:**

The online version of this article (10.1007/s00399-021-00770-7) contains supplementary material, which is available to authorized users.

## Abstract (de)

Die arrhythmogene Kardiomyopathie (AC) ist eine seltene Herzmuskelerkrankung mit genetischem Hintergrund und autosomal-dominantem Erbgang. Die AC ist charakterisiert durch ventrikuläre Arrhythmien (VA), Herzinsuffizienz (HF) und ein erhöhtes Risiko des plötzlichen Herztods (SCD). Schwangerschaften bei jungen Patientinnen mit AC stellen eine besondere Herausforderung mit hoher Bedeutung für die Lebens- und Familienplanung betroffener Frauen dar. Neben genetischen Mechanismen mit ihrem Einfluss auf die komplexe Pathophysiologie der AC belegen experimentelle und klinische Daten den pathogenetischen Zusammenhang von Ausdauerbelastung und Leistungssport mit dem frühzeitigen Auftreten und der raschen Progression von Symptomen und Komplikationen der AC. Schwangerschaft und Leistungssport teilen einige physiologische Anpassungsvorgänge. Bei AC führen beide zu ventrikulärer Volumenbelastung und myokardialen Dehnungsreizen. Daher wurde postuliert, dass bei AC Schwangerschaften mit einem erhöhten Risiko von HF, VA, und SCD sowie schwangerschaftsspezifischen Komplikationen einhergehen. Diese Hypothese konnte jedoch bislang nicht belegt werden. Bei der Mehrzahl der Frauen mit AC werden Schwangerschaften gut toleriert und verlaufen unauffällig. Schwangerschaftsbedingte Symptome (VA, Synkope, HF), und peripartale Komplikationen treten bei AC nicht häufiger auf, die Mortalität ist nicht erhöht. Auch die Anzahl vollendeter Schwangerschaften korreliert nicht mit einem früheren Auftreten oder beschleunigten Verlauf pathologischer Veränderungen oder einem erhöhten Risiko von VA oder HF während der Schwangerschaft oder im postpartalen Verlauf. Zusammenfassend bestehen somit bei Frauen mit AC keine medizinischen Argumente, von einer Schwangerschaft abzuraten. Voraussetzungen sind eine stabile Ausgangssituation von Rhythmus und Hämodynamik sowie eine multidisziplinäre Mitbetreuung und klinische Verlaufskontrolle während und nach der Schwangerschaft. Zudem sollte jeder Patientin mit AC, ihrem Partner sowie der Familie vor einer Schwangerschaft eine genetische Beratung angeboten werden.

## Introduction and epidemiology of AC

Arrhythmogenic cardiomyopathy (AC) is characterized by progressive myocyte cell death causing fibrofatty replacement of the myocardium, thus forming an arrhythmogenic substrate that predisposes to ventricular arrhythmias (VA) and sudden cardiac death (SCD) in young individuals and athletes [4, 16, 37, 59]. The disease is classified as a genetically determined primary cardiomyopathy [24, 40] based on mutations in desmosomal genes encoding for proteins responsible for mechanical cell-to-cell junction, integrity, and interaction [25].

Among other mechanisms that influence this complex pathophysiology, mechanical stress and stretch of ventricular walls have been demonstrated to contribute as a triggering factor to a more accelerated course of AC. Experimental [26, 51] and clinical data [28, 35, 52, 63] have emphasized and confirmed the pathogenetic role of strenuous exercise and competitive sports activity for the age-related penetrance with early onset and rapid progression of symptoms, the risk of VA, and the outcomes of heart failure (HF) in carriers of AC-associated desmosomal gene variants.

Among other hemodynamic alterations, pregnancy results in ventricular volume overload and myocardial stretch comparable with that observed in athletes. Similarly, pregnancy has been postulated as a potential risk factor for VA and SCD, as well as myocyte cell death, progression of structural ventricular deterioration, and HF [13]. However, this hypothesis has not been confirmed by the available evidence.

This review article aims to summarize the data and evaluate the published evidence on the acute and long-term risks of obstetric and maternal outcomes of single or multiple pregnancies in women with a clinical diagnosis of AC.

## Terminology and pathophysiology of AC

In AC, the right ventricle (RV) is usually affected earlier and more severely, as illustrated by the initial terms *“*(*arrhythmogenic) right ventricular dysplasia* *=* *ARVD”* [37, 43] or *“arrhythmogenic right ventricular cardiomyopathy* *=* *ARVC”* [4, 16, 38, 59]. Current knowledge, however, indicates that left ventricular (LV) involvement, either in late stages of advanced right-dominated AC or as a primary left-dominated manifestation early in the course of AC, is more frequent than previously estimated and diagnosed [18, 42]. For this reason, the more general term *“arrhythmogenic cardiomyopathy* *=* *AC”* has been introduced and is adopted to refer to the entity in this review article [17, 24, 60].

Over the past decade, rapid advances in molecular and clinical genetics have added significantly to the pathophysiologic understanding of AC [64], which is currently considered a “disease of the desmosomes” and intercalated discs. Following the *“final common pathway”* concept [60], various pathogenetic mutations in genes encoding for desmosomal cell-to-cell junction proteins (plakophilin‑2, desmoplakin, desmoglein‑2, desmocollin‑2, plakoglobin) or those that interact with them as binding partners (i.e., lamin A/C, desmin, THEM43, phospholamban), result in the same pathomorphological and arrhythmogenic phenotype [58, 60]. This is characterized by intercalated disc disruption and desmosomal loss, damage and/or dysfunction, and subsequent myocyte atrophy with fibrofatty replacement [51]. The resulting major clinical features of AC include:

1. *Ventricular arrhythmias (VA) *due to the arrhythmogenic substrate, mainly with areas of slow conduction and dispersion of refractoriness as a prerequisite for reentrant ventricular tachycardia (VT) or ventricular fibrillation (VF), and SCD
2. *Heart failure (HF)* due to regional loss of myocardium with subsequent replacement by scar tissue, thus resulting in regional and/or global RV and/or LV dysfunction [17, 18, 24, 38, 64].

## Epidemiology of pregnancy in AC

In Western countries, cardiovascular disease (CVD) is the leading cause of maternal mortality in the context of pregnancies. Compared with the low 0.007% case fatality rate of pregnancy in healthy women, mortality is much higher (approximately 1%) in women with CVD, ranging from 0.6 to 3.9% in developed versus developing countries. The majority of lethal outcomes of pregnancy in the mother is related to acute myocardial infarction, congenital heart disease, and cardiomyopathies [47, 72]. Although cardiomyopathies constitute only a small subgroup within the diversity of CVD, they represent severe causes of increased mortality (2.4%) and cardiovascular complications during pregnancy [47, 72].

Among the broad spectrum of heart muscle diseases, AC represents a rare entity that manifests in adolescence and early adulthood with signs and symptoms of VA and HF. The prevalence of AC has been estimated to be in the range of 1:5000, with significant geographical variations. Recent reports indicate that in AC, VA and disease progression can be worsened by vigorous exercise [26, 35, 51]. Pregnancy might be regarded as a state comparable to exercise. In addition to sympathetic stimulation, hormonal changes, and volume expansion, a rise in stroke volume and heart rate result in prolonged hemodynamic stress and increased wall stretch [13, 15, 53]. Therefore, theoretical concerns on pregnancy outcomes relate to acceleration of disease progression (VA, HF), resulting in pregnancy-related complications and increased maternal mortality. As AC is frequently diagnosed in women of childbearing age, data regarding the effect of pregnancy on progression of AC are needed to improve patient and family advice [13].

## Hemodynamic adaptations to pregnancy

In healthy women, pregnancy induces adaptations of cardiovascular hemodynamics and metabolism to meet the increased demands of the growing fetus and to prepare the mother for delivery. At 32 weeks of gestation, cardiac output reaches a maximum of up to 80% above baseline. Three quarters of this increase occurs by the end of the first trimester and is achieved by a rise in stroke volume in the first half of pregnancy and by an acceleration of heart rate thereafter. Ventricular volumes increase by 30%, whereas systolic RV and LV function are preserved. In addition, preload blood volume rises to 40% above baseline and systemic vascular resistance decreases during gravidity. These physiological adaptations result in biventricular dilatation and eccentric LV hypertrophy during pregnancy and reverse within approximately 6 months postpartum. During labor, cardiac output augmentation occurs due to uterine contractions, pain, and anxiety. Cardiac output increases by 80% immediately after delivery due to autotransfusion and then rapidly decreases within 10 min and returns to pre-labor values within 24 h [47, 53].

In patients with AC, RV and LV adaptation to pregnancy can be abnormal when the hemodynamic state is (partly) decompensated. Enhanced volumes and ventricular dilatation result in increased stretch of the RV and/or LV walls, with the potential to induce myocardial damage through disruption of cell-to-cell junctions in genetically predisposed pregnant women with AC. Strenuous exercise and competitive sports have been identified as triggering factors for stretch-induced aggravation of AC [35, 51]. Pathophysiological considerations imply that similar mechanisms may accelerate progression of AC also during pregnancy. Furthermore, advanced cardiac dysfunction in the mother may result in reduced uteroplacental flow and impaired fetal outcome [47].

## Preconception management

### Clinical evaluation.

All women with suspected or diagnosed AC who are considering pregnancy should undergo preconceptional clinical evaluation with detailed (family) history, electrocardiography (ECG), and initial transthoracic echocardiography. Upon further confirmation of AC, pre-pregnancy genetic counseling and additional diagnostic tests (i.e., exercise testing, more detailed echocardiography and cardiovascular magnetic resonance = CMR) should be supplemented by a cardiologist with expertise in managing women with AC and pregnancy [47, 72].

### Risk assessment.

High-risk patients identified by initial clinical risk assessment [10, 11, 19, 65] should be monitored throughout pregnancy by a dedicated multidisciplinary team of experts [47] with individualized coverage of all relevant medical fields (i.e., family medicine, internal medicine, general and interventional cardiology, electrophysiology, clinical genetics, heart failure, anesthesiology, gynecology, obstetrics, pediatrics, and psychology). To keep risks as low as possible for both the mother and the unborn child, cardiovascular interventions and medical treatment adaptations to optimize clinical stability should be scheduled prior to pregnancy whenever feasible [47, 72].

### Contraception.

Effective contraception is usually advised in young women with a clinical or genetic diagnosis of AC until genetic counseling has been completed. Due to the comparatively uneventful and benign course of most pregnancies in AC, there is no general concern that would discourage pregnancy or even argue for an early termination of gravidity in an otherwise healthy woman with AC. The decision for birth control and the choice of contraceptive measures follows the personal preferences of the woman and her partner rather than medical indications.

## Genetic counseling and testing

Arrhythmogenic cardiomyopathy (AC) is a familial heart muscle disease. Rare syndromic variants of AC with a cardio-cutaneous phenotype (Naxos and Carvajal syndromes) follow an autosomal recessive genetic trait with complete penetrance. However, the majority of AC patients have non-syndromic *“classical”* AC with an autosomal-dominant mode of transmission. Classical AC should not be considered as a simple monogenic disease, but rather as a complex genetic entity with both incomplete penetrance and variable expressivity [64]. Therefore, genetic counseling is mandatory in all index patients with AC and recommended in non-genotyped or affected family members [29, 58], particularly when genetic testing for pathogenic mutations causing AC is intended in the context of a planned pregnancy.

### Incomplete penetrance.

With an autosomal-dominant genetic mode of transmission, index patients with classical non-syndromic AC will transfer the pathogenic mutation to 50% of their offspring, irrespective of gender. However, due to incomplete and age-dependent penetrance, only 35% of family members and 60–75% of mutation carriers will ever manifest clinical features or symptoms of the disease, thus explaining the numerical difference between genetically and clinically affected relatives [29, 58, 60]. In addition, there is significant inter- and intra-familial variability in phenotypic expressivity and clinical manifestation [6, 21, 29, 58]. As a result, asymptomatic (“silent”) mutation carriers without clinical signs of AC are not uncommon. Although they frequently remain asymptomatic, they may transfer the mutation to the next generation, which may develop the full spectrum of disease expression.

### Molecular genetic technologies.

Next generation sequencing, whole-exome or whole-genome sequencing, and comprehensive cardiomyopathy-targeted multi-gene panel sequencing have heralded a new era of genetic diagnosis and genotype–phenotype correlations. Over the past decade, genetic testing has advanced to become a feasible, fast, and affordable procedure. It is now routinely used in the diagnostic assessment of cardiomyopathies (including AC), channelopathies, overlapping arrhythmia syndromes, and HF entities [60].

### Pathogenicity of genetic variants.

The detection of a genetic mutation or variant supports, but does not necessarily prove, a causative relation to penetrance, manifestation, expression, and severity of AC. Therefore, to draw any clinically relevant conclusions, positive genetic findings always require confirmation and evidence that the detected genetic variant is indeed pathogenic for AC [20]. Expert interpretation of gene–disease associations and variant pathogenicity is supported by the Clinical Genome (ClinGen) Resource Cardiovascular Working Group (https://clinicalgenome.org; [41, 45, 49, 60]). To determine the likelihood of pathogenicity of a genetic variant and its relation to disease, the following classification has been proposed: pathogenic (class 5: >95%), likely pathogenic (class 4: >90%), variant of uncertain significance = VUS (class 3: 10–90%), likely benign (class 2: <10%), and benign (class 1: <5%) [8, 20, 49, 60, 62].

### Timing of clinical re-evaluation.

In the case of detection of a (likely) benign mutation status (class 1 or 2) or a variant of uncertain significance = VUS (class 3) in relatives with ambiguous clinical findings, it is recommended to continue clinical re-evaluation at regular intervals [6, 58]. The timing of clinical follow-up visits should be scheduled at intervals according to the age-dependent penetrance of AC. In AC patients under the age of 10 years, clinical symptoms, VA, or sudden death are extremely infrequent [32] and are mainly associated with double (compound or digenic) or polygenic mutations [29, 50, 58, 71]. Therefore, non-invasive diagnostic re-evaluations (ECG and imaging techniques) should be scheduled starting from the age of 10 years and at intervals of 1–2 years (CMR at greater intervals). At ages above 25–30 years, follow-up intervals may be increased to 3–5 years [6, 58]. This covers the disease “hot phase,” when AC manifestation in adolescence and young adulthood is at its highest and overlaps with child-bearing age in women affected by AC. In contrast, new-onset AC beyond the age of 60 years is very uncommon [6, 29, 57]. Routine diagnostic screening may be stopped at this age in non-genotyped asymptomatic relatives and healthy mutation carriers [58]. Suspicious clinical symptoms or events should always trigger earlier re-evaluation.

In AC, electrical alterations may precede the detectable structural changes. For diagnostic re-evaluation, therefore, placing focus on ECG rather than imaging abnormalities has been proposed as long as the patient remains asymptomatic [6, 39, 56, 57]. Whenever asymptomatic individuals become symptomatic or present with new or progressive electrical abnormalities, cardiac imaging (echo, CMR) is recommended to assess the presence of structural damage and progression to clinical cardiomyopathy with a potential impact on risk assessment and treatment [56].

### Indications for genetic testing.

The following general aspects on the clinical utility of genetic testing in cardiomyopathies (including AC) are currently under discussion:

1. Molecular confirmation of a clinical diagnosis (possible, borderline, definite) in symptomatic patients (*confirmatory screening*)
2. Early detection and risk assessment of asymptomatic family members through systematic testing of the gene and (likely) pathogenetic mutation (class 4–5) detected in the genotyped proband (*cascade screening*)
3. Risk stratification of adverse events (VA, HF) during follow-up with an impact on treatment and management (gene- or mutation-specific risk)
4. Molecular differentiation of overlapping VA or HF syndromes
5. Differentiation of hereditary cardiomyopathy from acquired (non-genetic) disease
6. Research on the impact of genotype on pathophysiology, phenotype expression, and clinical course/risk stratification (approval by ethics committee required)

In AC, genetic testing is established only under particular clinical conditions, which should be discussed with the proband and the family during detailed genetic counseling before testing. The main indication for genetic testing in AC is *confirmatory testing* in the index patient. This should be followed by mutation-specific targeted *cascade screening* of family members, including testing of genetic variants with >90% pathogenicity (class 4 and 5) in the index patient with a confirmed diagnosis of AC.

In general, stand-alone confirmatory genetic testing of probands without an intention to subsequently perform cascade screening in the family is of low clinical impact. High expectations for a potential usefulness for risk stratification and prognostic evaluation were disappointing since no “malignant” genes or mutations were identified. Given the incomplete penetrance and variable expressivity in AC, the clinical manifestation of the same mutation can be substantially different within the same family [6, 29, 58].

### Mutation-negative patient.

The inability to detect a (likely) pathogenic variant (class 4 or 5) in an index patient with a definite clinical diagnosis of AC does not necessarily allow exclusion of the disease [58]. This is due to the fact that 25–40% of patients with clinically confirmed AC still lack evidence of a currently identified pathogenic gene variant despite full genetic screening. In this particular situation, mutation-specific cascade screening is impossible and genetic testing of relatives, therefore, usually unhelpful [58]. However, index patients with AC but no detectable mutation appear to have a more benign course of AC with respect to higher ages at disease onset, clinical presentation, symptoms, sustained VA, and development of HF compared with index patients with an identified mutation [29].

### Evaluation of family members.

Although the rate of major clinical events was relatively low in family members with mutations [29], asymptomatic mutation carriers exhibit a six-fold increased risk of subsequent disease manifestation and expression of clinical AC compared to family members without evidence of a pathogenic variant [21, 58]. In those asymptomatic mutation carriers, closer clinical follow-up and a recommendation to discourage strenuous physical exercise and competitive sports appears justified in order to prevent, diminish, or postpone disease manifestation and acceleration. In contrast, if a pathogenic mutation is detected in the proband but excluded in a family member, this relative is not at risk for AC manifestation and dedicated follow-up investigations are not necessary.

In this situation, there remains only a small residual risk that the proband may carry an additional undetected or unidentified second pathogenic mutation with the potential for genetic transfer to future generations. To cover this issue, it is important to test the mutation-positive proband with sequencing for all known AC-causing pathogenic variants in order to detect a second or third “compound” mutation in the same gene or “digenic”/“polygenic” variants in other genes that would otherwise not be tested during cascade screening in the relatives [60, 71].

### Gene-dosing effect.

Compound heterozygosity and digenic or polygenic mutations are not infrequent in AC and support the concept of a *“gene-dosing effect”* to explain variability of penetrance, expressivity, and severity of disease manifestation [6, 60, 71]. In general, AC patients with double or multiple pathogenic mutations have a worse clinical outcome with respect to earlier onset of symptoms and first sustained VA, a higher incidence of sustained VT/VF and sudden death, as well as a more progressive course of symptoms, LV dysfunction, and HF compared to those carrying a single mutation [6, 58, 71]. These complex genetic mechanisms may be further modulated by additional genetic (i.e., “modifier genes,” polymorphisms) and environmental factors (i.e., competitive sports, pregnancy, viral exposure) [58, 71].

### De-novo mutations and founder effects.

Spontaneous de-novo AC-causing gene mutations are rare. “Founder effects” may explain the inhomogeneous geographical distribution of AC-causing mutations as well as the variable prevalence and severity (clinical manifestation) in heterogeneous AC populations [62]. Differences in the application of clinical diagnostic criteria may further add to such geographic disparities in the reported prevalence of AC [60].

### Genetic counseling.

For couples with one or both partners affected by AC and (likely) pathogenic AC mutations (class 4 or 5), such complex genetic information is of the utmost importance but may also cause marked anxiety. AC therefore requires professional genetic counseling with particular focus on individual family planning, contraception, and pregnancy, as well as the clinical management and follow-up intervals of asymptomatic and genetically affected children. As a rule, in difficult decisions during patient management, clinical judgement should generally be ranked higher than genetic findings.

The advantages of genetic testing in AC need to be balanced against its potential disadvantages, particularly with respect to the timing of testing in asymptomatic mutation carriers [2]. Marked anxiety and psychological disorders caused by life-long “disease-labeling,” as well as lifestyle restrictions, discouragement from sports activity, and potential limitations in social security and insurance are only a few examples illustrating the areas of caution. These issues are even more relevant in children, minors, and adolescents, since these individuals should have the opportunity to decide in a self-determined manner whether or not they desire genetic testing with all its inherent positive and negative aspects [2].

### Preimplantation genetic diagnostics

Genetic testing can also be performed in the unborn offspring of a genotype-positive patient or mutation carrier using confirmatory screening in the adult proband and subsequent mutation-specific cascade screening with preimplantation diagnostics (PID) during the early embryonic development of the offspring. Preimplantation genetic diagnostics is an adjunct to assisted reproductive technology and requires in-vitro fertilization (IVF) to obtain oocytes or embryos for the evaluation of pathogenic mutations (i.e., AC). The evidence of pathogenicity must be strong (>95%; class 5) to ensure that test results are reliable.

Genetic profiling in the oocyte or embryo prior to implantation for the purpose of prenatal diagnosis of genetic diseases is technically feasible but discussed with intense controversy and ethical concerns, a discussion that is clearly beyond the scope of this review article.

## Drug therapy during pregnancy

During the period of pregnancy and lactation, women with pre-existing AC may require continued or extended drug treatment directed toward arrhythmias and/or HF. Attention should be paid to symptoms of decompensated HF to rule out pregnancy-induced acceleration of AC or superimposed peripartal cardiomyopathy (PPCM). Aggravating factors, such as electrolyte imbalance, hyperthyroidism, and anemia require correction.

During pregnancy, drug pharmacokinetics are significantly affected due to changes in absorption, distribution, biotransformation, and excretion. Increased activity of enzyme systems, glomerular filtration rate, and plasma volume, as well as changes in protein binding and reduction of serum albumin levels contribute to altered pharmacokinetics. Venous congestion of the liver compromises metabolism of physiological and pharmacological substrates, giving rise to undue lowering or elevation of drug concentrations. Unwanted drug interactions might even result in toxic or teratogenic effects in the fetus (passage of placenta) or newborn (passage to breast milk).

For an assessment of drug efficacy and safety during pregnancy and breastfeeding, prospective randomized clinical trial data from pregnant women with AC are lacking. As a general rule, most drugs should be strictly avoided in the first trimester of pregnancy (particularly during the first 8–10 weeks of gestation), when teratogenic and fetotoxic risks are highest.

In pregnant women with AC, drug therapy should be limited to strong maternal indications, and any necessary drug should be administered at the lowest recommended dose. However, in the case of an emergency, drugs not recommended by the pharmaceutical industry during pregnancy and breastfeeding should not be withheld from the mother. In this difficult bailout situation of “off-label” drug therapy, potential adverse or teratogenic effects on the fetus must be weighed against the beneficial and potentially life-saving maternal indication [47, 72]. Further advice regarding drug treatment in pregnancy is provided on internet databases, accessible on http://www.embryotox.de or http://www.safefetus.com.

### Treatment of heart failure

Pregnant women with AC and HF during pregnancy and postpartum should be treated according to current guidelines for acute and chronic HF in non-pregnant patients, respecting the indications for device implantation and pregnancy-related contraindications for some drugs.

***Inhibitors of the renin–angiotensin–aldosterone system (RAAS)***, such as angiotensin-converting enzyme (ACE) inhibitors, angiotensin II receptor blockers (ARB), sacubitril/valsartan, direct renin inhibitors, and mineralocorticoid receptor antagonists (MRA) are contraindicated due to potential fetotoxicity. Nitrates can be used instead of RAAS inhibitors for vasodilation and afterload reduction if needed [47, 72].

***Beta-blockers*** are generally indicated in patients with HF due to their antiadrenergic and antiarrhythmic effects. In most patients, they are well tolerated, although a decrease in fetal heart rate is frequently observed. β_1_-selective beta-blockers, such as metoprolol or bisoprolol, are preferred over non-selective atenolol. Neonates should be monitored for 24–48 h after delivery to exclude hypoglycemia, bradycardia, and respiratory depression [47, 72].

***Diuretics*** such as hydrochlorothiazide, torasemide, or furosemide should only be used in pulmonary congestion since they may decrease blood flow over the placenta. Digoxin may be used safely during pregnancy. Measurements of blood serum levels, however, are subject to errors during pregnancy and are therefore not helpful for dose titration [47, 72].

It is rare that women with AC and new onset of severe RV and/or LV dysfunction during pregnancy need to be advised against further pregnancies out of concern about a further deterioration of cardiac function. However, women with AC and severe RV dysfunction or biventricular HF before pregnancy should be given clear information about the increased risk of pregnancies.

### Treatment of arrhythmias

Arrhythmias requiring antiarrhythmic drug treatment develop in up to 15% of pregnant women with structural heart disease. New onset of symptomatic arrhythmias during pregnancy is rare and should prompt investigations to rule out structural abnormalities or aggravation of ventricular dysfunction (i.e., AC or dilative cardiomyopathy).

#### Supraventricular arrhythmias.

In pregnant women with AC, the prevalence of paroxysmal supraventricular tachycardia (SVT), atrial tachycardia, and atrial flutter or fibrillation is low.

SVT is usually well tolerated but may become more frequent, refractory, and symptomatic during pregnancy. First-line treatments for the termination of acute paroxysmal SVT during pregnancy include vagal maneuvers and intravenous bolus administration of adenosine. If this fails, metoprolol or verapamil may be alternative options during pregnancy. Flecainide or sotalol should only be options for more complex and highly symptomatic atrial arrhythmias. However, due to their proarrhythmic effects, they are not recommended in patients with relevant structural heart disease, which should be excluded prior to use. Digoxin has a limited role and may be used to facilitate rate control of atrial arrhythmias as a stand-alone or adjunct treatment supportive to beta-blockers or flecainide [47, 72].

#### Ventricular arrhythmias.

In the acute situation, direct-current cardioversion is suggested as a first-choice treatment for all sustained VA with hemodynamic compromise. It can be safely performed at all stages of pregnancy without adverse effects to the fetus, at least beyond the 4th–6th week of gestation [9].

Long-term treatment of VA with antiarrhythmic drugs during pregnancy should be used with caution and is indicated only if symptoms are intolerable or if VA causes hemodynamic compromise. In pregnant women with AC and hemodynamically stable VA, oral β_1_-selective ***beta-blockers*** such as metoprolol or bisoprolol are recommended as first-choice drugs to reduce the sympathetic drive and thereby reduce symptomatic premature ventricular contractions (PVCs) and sustained VT episodes. They are considered safe during pregnancy, particularly after the first trimester. Long-term use of atenolol has been associated with intrauterine fetal growth retardation and a higher prevalence of preterm delivery. These were mainly observed in newborn babies when the mother received atenolol in the first trimester, which is therefore discouraged.

***Sodium channel blockers*** such as *flecainide* are a second choice during pregnancy. In the absence of advanced structural heart disease, flecainide is well tolerated and reasonably safe for the mother and child. No teratogenic effects have been reported in the literature. Since placenta transition is good (particularly during the third trimester), fetal arrhythmias may be treated by giving medication to the mother. Other sodium channel blockers (*propafenone, mexiletine, disopyramide*) should be used with caution, since there is less experience in pregnant women, particularly those with AC [47, 72].

***Sotalol*** has combined beta-blocking and class III antiarrhythmic properties and is frequently effective for the treatment of symptomatic VA in AC [65, 66]. However, there is only limited experience with this drug during pregnancy and caution is advised concerning its potential for drug-induced QT prolongation and torsade des pointes in both mother and child.

***Amiodarone*** should be avoided for chronic treatment whenever possible in pregnant women due to its potential for severe multi-organ side effects (thyroid, lungs, liver, and eyes) in the mother and fetus during long-term treatment. Amiodarone should only be used in selected high-risk pregnant patients with AC and symptomatic VA resistant to other therapies [65, 66]. For *dronedarone* and *dofetilide*, there is no experience in pregnancy, particularly in AC.

Other drugs with antiarrhythmic potential, such as *digoxin, adenosine, or verapamil*, are considered safe during pregnancy. However, they are usually not effective for the treatment of VA since they occur in AC [47, 72].

## Devices and interventional procedures during pregnancy

Catheter ablation and implantation of pacemakers or defibrillators (ICD) are feasible during pregnancy [31]. However, when such invasive procedures are performed during pregnancy, increased risks for mother and child must be accepted. Therefore, to outweigh the elevated risks, indications must be restricted to highly selected patients with severe symptoms from recurrent or life-threatening VA.

### Radiation exposure.

Strict caution should be ensured to keep radiation doses “as low as reasonably achievable” (= ALARA concept). Radiation risk to the fetus varies depending on dose, duration of exposure, and gestational age of the fetus. Measures taken to reduce radiation exposure to the fetus include [47, 72]:

- Echocardiography for guidance whenever possible
- Non-fluoroscopic approach with electroanatomic three-dimensional (3D) mapping
- Fluoroscopy time as short as possible
- Only low-dose or pulsed fluoroscopy
- Projections with low radiation (i.e., anteroposterior)
- Placement of the radiation source distant and the detector close to the patient
- Tight collimation to the area of interest
- Avoidance of direct radiation of the abdominal and pelvic regions
- Experienced interventional cardiologist as investigator

In the majority of diagnostic medical procedures, the radiation dose to the fetus is below 1.0 mGy. The risk of miscarriage, major malformations, or malignancy is low in fetuses exposed to 50 mGy or less. The teratogenicity of radiation is dose-dependent, with the risk of fetal malformation increasing significantly at fetal doses above 100–150 mGy [47, 72]. Most interventional procedures do not expose the fetus to anywhere near such high levels of radiation. For example, coronary angiography results in an estimated fetal radiation exposure of 1.5 mGy, percutaneous coronary interventions (PCI) or catheter ablation in an exposure of 3–5 mGy. Since the carcinogenic risk of ionizing radiation follows a linear no-threshold risk model, radiation exposure in utero should remain an important concern. Whenever possible, procedures requiring fluoroscopy should be delayed until after pregnancy or at least until after the 12th–15th gestational week, when the fetus is less sensitive to radiation effects [47, 72].

### Pacemaker therapy.

In pregnant women with AC, bradycardia usually implies no fetal or maternal complications. Sinus bradycardia may occur during the final period of pregnancy, including delivery, and is most frequently attributed to the supine hypotensive syndrome of pregnancy, which is managed accordingly by changing the position of the mother to a left lateral position. Low-degree atrioventricular block (AVB) and isolated congenital complete AVB are highly uncommon in AC, with favorable outcome throughout pregnancy, usually without progression of conduction delays or symptoms. Permanent pacemaker therapy is very rarely indicated and can be performed, if required, with little or no radiation exposure when modern techniques are used.

### Implantable cardioverter defibrillator (ICD).

The indications to implant an ICD in pregnant women with AC follow established recommendations from Task Force Consensus reports on AC [18, 19, 24, 67]. Risk stratification should identify high-risk candidates with AC who would benefit from prophylactic ICD implantation [10, 11, 19, 65] before, after, and even during pregnancy [9, 23, 31, 44, 46, 55]. Since in pregnant women with AC, ICD implantation requires a strong indication, only scant data are available and little is known about appropriate or inapprorpriate ICD discharges on fetal, neonatal, or maternal outcomes.

Transvenous ICD systems carry a long-term risk of RV defibrillation lead failure (undersensing, oversensing due to lead fracture or insulation defects). Undersensing as a specific problem in AC results from malposition of the RV lead in areas of primary or progressive severe RV myocardial damage, independent of pregnancies [67].

Subcutaneous ICD systems without the need for transvenous leads may be an alternative option in pregnant women with AC. They have been reported to be safe with high efficacy of appropriate ICD shocks and acceptable complication rates, mainly attributed to T‑wave oversensing with inappropriate ICD shocks [44]. Both transvenous and subcutaneous ICD systems have been implanted successfully in patients with AC after delivery or (in emergencies) also during pregnancy [23, 31, 46]. No major complications have been reported, with only minimal or no radiation exposure from fluoroscopy.

Studies looking at the maternal safety of ICD therapy during pregnancy found no evidence to suggest that carrying an ICD in AC in itself poses a contraindication to future pregnancies [9, 31, 46]. Therefore, ICD implantations are considered safe and effective during pregnancy in women with AC and in their offspring. In the fetus, cardioversion shocks from external or implanted defibrillators have been shown to be without adverse events, at least after the 4th–6th week of gestation. However, Boulé et al. [9] reported a questionable case of miscarriage, which was potentially attributable to an ICD shock within the first 4 weeks of gestation. The authors concluded that an “all-or-nothing” law might have caused this unusual adverse event during very early pregnancy. Fetal monitoring is advised since transient fetal arrhythmias may occur in the context of cardioversion or defibrillation. These recommendations are applicable to external defibrillation shocks as well as internal shock delivery from an implanted ICD.

### Other devices.

Indications for implantation of more complex devices such as cardiac resynchronization therapy (CRT) or cardiac contractility modulation (CCM) to improve advanced heart failure generally follow current HF guideline recommendations. However, they should preferably be implanted within a time frame before or after pregnancies to avoid surgical or arrhythmic complications and radiation exposure.

### Catheter ablation.

In selected women with AC, catheter ablation has been reported as a therapeutic option for the treatment of frequent and drug-refractory VA during pregnancy. Patients with localized manifestation of AC and only one dominant arrhythmic focus have been reported to have excellent long-term results and prognosis [12]. In contrast, those patients with diffuse or extensive AC and multiple VT origins in the RV and LV have lower acute success rates, and new arrhythmic foci appear with structural disease progression during the long-term course of AC [65]. Hormonal or autonomic imbalance (as present in pregnancy) may increase the risk of frequent VT recurrences (VT storm) in AC. In line with these observations, a large single-center registry of patients with VT ablation for electrical storm reported a 14% prevalence of AC among patients with organic heart disease and an implanted ICD [12].

For the treatment of hemodynamically unstable, frequently recurring sustained VT otherwise refractory to electrical cardioversion (incessant VT or VT storm), current guidelines recommend antiarrhythmic drug therapy with metoprolol, sotalol, or amiodarone. If medication fails, bailout or emergency catheter ablation during pregnancy has been performed successfully in women with AC [55]. In such urgent situations during pregnancy, VT ablation in AC can be performed with minimal or no radiation by using non-fluoroscopic catheter techniques and electro-anatomical 3D mapping systems, thereby minimizing radiation exposure to both the mother and the unborn child [14, 55].

## Breastfeeding in AC

There is no contraindication to breastfeeding in women with AC or CVD in general. Of note, most beta-blocking agents are taken up by the infant during breastfeeding. This requires surveillance of the neonate concerning bradycardia and hypoglycemia. Of importance, other drugs (i.e., renin–angiotensin–aldosterone system [RAAS] inhibitors, amiodarone) may cause severe adverse effects through excretion to the mother’s milk and subsequent transfer to the infant. If such drugs are urgently required during the period of breastfeeding, early delactation with dopamine receptor agonists (i.e., bromocriptine 2.5 mg twice daily) may be necessary [46, 71].

## Outcomes of pregnancy in women with AC

Since evidence from controlled randomized trials is lacking in this field, most of the current management recommendations and strategies for pregnancy in AC are based on observational and non-randomized studies or data derived from registries or expert consensus. Therefore, only scarce data are available to guide cardiologists and obstetricians.

Only case reports [1, 3, 15, 22, 23, 30, 33, 34, 54, 61] and small series [5, 7, 9, 36] with a total of 30 pregnancies in 30 women with AC had been reported prior to the first larger case studies and registry reports on pregnancy in AC that were recently published ([13, 27, 31, 48, 69]; Tables 1 and 2). All reported pregnancies in AC were singleton. No twin pregnancies have been published in AC so far.

**Table 1:** Obstetric, fetal, and neonate outcomes

First author	Year	Women	Pregnancies	Live birth	Miscarriage	Cesarean section	Preterm delivery	Birth weight
		(*n*)	(*n*)	(*n*)	(*n*; %)	(*n*; %)	(*n*; %)	(mean g)
Bauce [5]	2006	6	6	6	0	4 (67)	0	3490 g
Boulé [9]	2014	2	2	2	0	1 (50)	1 (50)	NR
Hodes [31]	2016	26	39	39	6 (15)	11 (28)	2 (5)	3400 g
Billebeau [7]	2018	3	3	3	0	0	0	NR
Gandjbakhch [27]	2018	23	60	50	10 (17)	8 (13)	2 (3)	3229 g
Castrini [13]^a^	2019	58	88	86	2 (2)	6 (7)	NR	NR
Luo [36]	2020	9	9	9	0	4 (44)	0	3118 g
Wu [69]	2020	120	224	192	32 (14)	44 (20)	10 (5)	NR
Platonow [48]	2020	120	261	261	NR	NR	NR	NR
*n*		*367*	*692*	*648*	*50/431*	*78/431*	*15/343*	*104/648*
*%*		*–*	*–*	*–*	*11.6*	*18.1*	*4.4*	*16*
*g*		*–*	*–*	*–*	*–*	*–*	*–*	*3292*

*mean g* Mean grams, *NR* not reported

^a^Reference [48] includes 58 patients

**Table 2:** Maternal outcomes

First author	Year	Women	Pregnancies	Nulliparous	Age 1st pregn	Follow-up	Mortality	SCD	Syncope	VA	HF	HTx
		(*n*)	(*n*)	(*n*)	(yrs)	(yrs)	(*n*; %)	(*n*; %)	(*n*; %)	(*n*; %)	(*n*; %)	(*n*; %)
Bauce [5]	2006	6	6	0	29	2.6	0	0	0	1 (17)	0	0
Boulé [9]	2014	2	2	0	31	0.5	0	0	0	0	1 (50)	0
Hodes [31]	2016	26	39	58	31	6.5	0	0	0	5 (13)	2 (5)	0
Billebeau [7]	2018	3	3	0	34	NR	0	0	NR	1 (33)	0	0
Gandjbakhch [27]	2018	23	60	0	30	NR	0	0	2 (3)	2 (3)	0	0
Castrini [13]^a^	2019	58	88	19	29	19	2 (3)	0	15 (17)^b^	22 (25)^b^	NR	2 (3)
Luo [36]	2020	9	9	0	31	NR	0	0	0	0	0	0
Wu [69]	2020	120	224	37	25	8.0	36 (23)^b^	2 (2)	2 (1)	1 (0.5)	1 (0.8)	0
Platonow [48]	2020	120	261	79	25	2.0	5 (4)	2 (2)	14 (5)	2 (0.8)	0	5 (4)
*n; mean*		*367*	*692*	*193*	*26.5*	*4.4*	*43*	*4*	*33*	*34*	*4*	*7*
*% women*		*–*	*–*	*–*	*–*	*–*	*11.7*	*1.1*	*8.8*	*9.3*	*1.3*	*1.9*
*% pregnancy*		*–*	*–*	*–*	*–*	*–*	*6.2*	*0.6*	*4.8*	*4.9*	*0.7*	*1.0*

*Age 1st pregn.* age at first pregnancy, *HF* Heart failure, *NR* not reported, *HTx* heart transplantation, *SCD* sudden cardiac death, *VA* ventricular arrhythmias

^a^Reference [48] includes 58 patients

^b^Higher event rates are caused by very long intervals between last pregnancy and diagnosis of arrhythmogenic cardiomyopathy (see text for details)

### Obstetric outcomes: induction, labor, and delivery

Data on obstetric, fetal, and pediatric outcomes of pregnancies in women with AC are summarized in Table 1. There were 648 live births from 692 pregnancies in 367 women with AC who were included into the analysis of nine studies with published data on pregnancies in AC. Single case reports were not included.

#### Timing and conditions of delivery.

The ideal timing for spontaneous delivery is after 37 weeks in women with stable heart rhythm and hemodynamics and fetal well-being. In decompensated, unstable, or uncertain maternal conditions, induction of labor and planned delivery under optimal conditions is the preferred option.

#### Miscarriage and abortion (≤ 13 weeks’ gestation).

Fetal miscarriage or abortion ≤ 13 weeks of gestation occurred in 50 of 431 (12%) women with AC summarized in this review. Almost all of these were spontaneous abortions. One study reported two stillbirths [13]. This rate of miscarriage lies within the normal range reported in the background population.

#### Preterm delivery and low birth weight.

Calculated from the published data from pregnancies in AC, preterm delivery occurred in 15 of 343 live births (4.4%) and therefore within normal limits. The mean birth weight reported from 114 neonates was 3292 g (Table 1). Patients on treatment with beta-blockers had lower birth weights [5, 7, 9, 27, 31, 35]. Atenolol, which is also known to cause intrauterine growth retardation, was very uncommonly used in the setting of pregnancy in AC.

#### Cardiovascular maternal and fetal monitoring.

Monitoring of heart rhythm and blood pressure is essential in all women with AC during the peripartal period due to the potential occurrence of VA during labor and severe hemodynamic fluctuations due to large volume shifts in the immediate postpartum period. In patients with AC, peripartum surveillance requires a multidisciplinary team of experts in the fields of family medicine, general and interventional cardiology and electrophysiology, gynecology and obstetrics, pediatrics, anesthesiology and intensive care medicine, clinical genetics and genetic counselling, as well as other specialists upon request. Invasive monitoring is very rarely required.

#### Labor induction.

Labor includes induction, treatment of pain, and assisted delivery. Prolonged labor should be avoided in women with AC. Induction with prostaglandins should be used with caution since it poses the risk of blood pressure fluctuations, coronary vasospasm, and arrhythmias. In AC patients, particularly those without an ICD implanted, a defibrillator should always be nearby for immediate use in the case of occurrence of a potentially life-threatening sustained VA [47, 72]. During labor, the mother should be placed in a left lateral position to minimize blood pressure fluctuations. Maternal pushing can be diminished by delivery assisted by forceps, vacuum extraction, and effective analgesia [47, 72]. Epidural anesthesia is recommended to reduce pain-related sympathetic activity as a potential trigger of VA in the mother affected with AC.

#### Vaginal delivery or cesarean section.

In women with AC, like in most other CVD and cardiomyopathies, vaginal delivery is preferred over cesarean section in women with AC due to its lower risk profile and complication rates, particularly related to thromboembolism as well as major bleeding and blood loss in women on anticoagulant treatment. Therefore, cesarean section delivery should be reserved mainly for obstetric indications or preterm labor, as well as for women presenting on effective oral anticoagulation at the time of labor [47, 72].

The majority of pregnant women with AC underwent vaginal delivery. Cesarean section was performed in 78 of 431 (18%) women with AC summarized in this review (Table 1), with <10% being for maternal medical reasons and >90% for obstetric indications. Hodes et al. [31] reported cesarean sections in 11 of 39 women with AC (28%), all but one (HF) for obstetric indications. Another large study recently published by Wu et al. [69] reported similar results with cesarean section in 44 of 224 Chinese women (20%). Castrini et al. [13] and Gandjbakhch et al. [27] both reported lower rates of cesarean sections in their cohorts (7 and 13%, respectively). However, all rates of cesarean sections are within normal ranges for a given socio-economic region. No thromboembolic or bleeding complications were reported for elective cesarean sections in patients with AC.

### Maternal and offspring outcomes

Data on maternal outcomes of pregnancies in women with AC are summarized in Table 2. There were 648 live births from 692 pregnancies in 367 women with AC and a mean age of 29 years at first pregnancy that were included in the analysis of nine studies with published data on pregnancies in AC. Single case reports were not included. In 171 of 231 patients (74%) in whom genetic testing was reported, a pathogenic mutation was detected. In the control group (from four studies), 193 nulliparous women with AC were compared with the pregnant cohort of 324 women. The results demonstrated similar event rates for mortality, SCD, aborted SCD, VA, and HF in both groups. However, these results are prone to selection bias since women in the control group with younger age at symptom onset and more severe AC manifestation may have been advised against pregnancy.

#### Maternal mortality and complications (VA, HF).

In the majority of women with AC, the course of pregnancy and postpartum, as reported in the available studies, was uneventful with regard to pregnancy-related mortality and complications. Depending on the number of women enrolled in the study, the individual risk profile, and the duration of follow-up, the rates of maternal all-cause death varied from 0 to 4%. The occurrence and number of pregnancies had no impact on mortality, heart transplantation, or the incidence of maternal complications (VA, HF) [31, 48].

In all, 33 of 241 women (14%) reported a worsening of symptoms during 425 pregnancies (8%). The event was defined either as a new onset of sustained VT, VF, syncope, or frequent PVC, or as an increase in PVC numbers by more than 100%.

Hodes et al. [31] reported data from the combined AC registries of the Johns Hopkins Hospital (Baltimore, USA) and the Dutch Interuniversity Institute (Netherlands). The authors reported on 26 women with diagnosed AC and 39 pregnancies >13 weeks of gestation. At last follow-up (mean, 6.5 years), all women with AC after pregnancy were alive and without the need for cardiac transplantation. Acceleration of disease progression with HF was observed in two women (5%), both with pre-existing right or biventricular dysfunction and both with successful outpatient management without the need for hospitalization. There were five pregnancies complicated by single episodes of sustained VT (13%) without an increased likelihood of experiencing a first sustained VA during pregnancy [31]. In women with AC, new onset or acceleration of VA occurred in less than 10% of pregnancies. Pregnancy-related VA were primarily related to the phenotypical severity rather than pregnancy itself.

Platonov and Castrini et al. [13, 48] recently published similar results in their latest series from the Scandinavian Nordic Registry, including 261 pregnancies in 120 women with AC. They reported two maternal SCD, five aborted SCD, five heart transplantations, and a total of 41 events during long-term follow-up. However, in only two of 120 women or two of 261 pregnancies (1.7 and 0.8%, respectively) did sustained VT occur during pregnancy, in another five patients within 2 years after completed pregnancy [48]. One patient experienced deep vein thrombosis, complicating the postpartum course [13]. The cumulative event rates during the long-term follow-up were higher, reflecting the prolonged interval between pregnancy and established diagnosis of AC.

Both groups [13, 31, 48] found no difference in clinical event rates (including mortality, SCD, aborted SCD, heart transplantation, HF, and VA) compared with control groups of women with AC who had not given birth. In agreement with others [27], both reported no evidence for acceleration of VA and HF, either during pregnancy or early or late after childbirth. They concluded that pregnancy was not the driving force for disease progression in AC.

Similar results were recently published by Wu et al. [69], who analyzed 224 pregnancies in 120 patients including 30 spontaneous and two medical abortions. In all, 12 cardiac adverse events were recorded during pregnancy, including new-onset frequent PVC in three, increased previous PVC numbers by more than 100% in five, and syncope in two patients. Sustained VT and HF requiring hospitalization occurred in one patient each. In comparison with a control group of 37 nulliparous women with AC, there was no difference in the core outcome parameters.

In contrast to other publications, however, Wu et al. [69] reported an all-cause mortality rate of 23% (*n* = 36 of 120) during the long-term median follow-up of 8 (1–32) years. However, only two of 36 deaths were sudden, and none with a diagnosis of AC. The majority of deaths occurred very late after previous pregnancies. Risk factors for all-cause mortality included earlier age at first symptom onset and decreased left ventricular ejection fraction (LVEF), whereas pregnancy did not influence the long-term survival. The study cohort differed significantly from other publications by a much longer follow-up and a substantially higher risk profile, including a history of cardiac arrest (40%), sustained VT/VF (74%), HF (33%), heart transplantation (17%), catheter ablation (50%), and ICD implantation (17%) [69]. Similar to the studies by Castrini and Platonov [13, 48], the majority of pregnancies (in 76% of women) occurred long before the diagnosis of AC was made.

#### Impact of multiple pregnancies on VA and HF.

Platonov et al. [48] retrospectively analyzed the course of 261 pregnancies in 120 women with at least one childbirth. Of these, 58 patients were previously reported [13]. They found that the number of prior completed pregnancies (one = 20, two = 67, three or more = 37 childbirths) was not associated with VA and HF risk. Although the number of pregnancies was related to increased RV outflow tract diameter in single variable analysis, this interaction was not confirmed when corrected for body surface area and age [48]. No other measure of cardiac structure or function was associated with the number of pregnancies. VA-free survival after the age of 45 years did not differ between those who gave birth and nulliparous women as a control group. Contrary to expectations, the nulliparous women had earlier onset of symptoms and more advanced electrical and structural disease compared to women with AC who had given birth at least once. The authors concluded that pregnancy-related VA was primarily related to the pre-existing phenotypical severity of AC rather than pregnancy itself [48].

#### Offspring mortality and complications.

The overwhelming majority of infants were born alive without obstetric complications and stayed healthy during long-term follow-up. No fetal or neonatal malformations were reported from pregnancies in AC, either with or without antiarrhythmic medication given to the mother. However, there were two stillbirths [13] and four cases of neonatal SCD [27, 69] without diagnosis of AC. In addition, there were three cases of sudden death during follow-up at 12, 19, and 22 years of age [27]. Only the latter male offspring was diagnosed with AC before sudden death and was found to carry the same desmocollin‑2 mutation as his affected mother. In the remaining cases of reported SCD, no AC was diagnosed.

#### Thromboembolic complications.

Pregnancy is a hypercoagulable state associated with an increased risk of thromboembolic complications. In AC, thromboembolic complications may result from intracardiac thrombus formation in ventricular aneurysms and sacculations due to RV and/or LV dysfunction. Wu et al. [70] reported the largest retrospective study on the prevalence, risk factors, and prognosis of intracardiac thrombi in patients with AC. They observed 10 ventricular thrombi in eight of 193 patients with AC (4%). Seven of 10 thrombi were located in the RV apex. Apart from atrial fibrillation, LV dysfunction and female gender appeared to be associated with a higher risk for intracardiac thrombosis in AC. Wlodarska et al. [68] reported similar results in their retrospective analyses in 126 patients with AC. During long-term follow-up of 99 ± 64 months, the annual incidence of ventricular thrombi was 0.5% per year. None of these patients were pregnant or had recently given birth to a child, either as vaginal delivery or as a cesarean section. Only one pregnant woman with AC reviewed in the present article experienced deep vein thrombosis [13]. Therefore, it appears that despite the general hypercoaguable state of pregnancy, women with AC do not carry a specifically enhanced risk of thromboembolic complications when pregnant.

## Résumé

Pregnancy shares some physiological adaptive mechanisms with exercise. In patients with AC, exercise-induced volume overload results in myocardial stretch, which promotes VA and HF by aggravating myocardial damage on the basis of a genetic predisposition with mutations in desmosomal genes [35, 51, 52]. Similar mechanisms have been hypothesized to accelerate VA and HF and to worsen the prognosis during or after pregnancy in patients with AC. However, the available experience and recent published evidence indicate that the effects of hemodynamic stress are more favorable during pregnancy compared with exercise.

Diverging mechanisms of chronic (adaptive) stretch to the myocardium during pregnancy as compared with strenuous exercise have been discussed to explain these differences. In contrast to exercise, the relatively shorter term and gradually developing nature of hemodynamic load during pregnancy might limit the adaptive remodeling process [31]. In AC patients, doses of harmful exercise or athletic activity range from 2 to 4 h per week for one to several years [35]. Pregnancy, however, is limited to a 9-month period with lower intensity of cardiac stress over shorter periods of time. In addition, pregnancy is characterized by a decrease in vascular resistance by approximately 30%, causing peripheral vasodilatation. In contrast, exercise induces an increase in afterload. Other speculations include hormonal and adaptive mechanisms involved during pregnancy that may be protective for the myocardium [13]. These hypotheses, however, require further confirmation from experimental and clinical investigations.

Taken together, the available evidence does not support the hypothesized association between single or multiple pregnancies and myocardial stretch-induced acceleration of AC with an increased risk of VA and HF. VA and HF during or after pregnancies are primarily related to the phenotypical severity of AC rather than pregnancy itself, which has not proven to independently worsen the prognosis and long-term course of AC.

## Practical conclusion

In most women with AC, pregnancy is well tolerated, uneventful, and has a benign course. Accordingly, there is no indication to advise against pregnancy in patients with AC, provided cardiac rhythm and hemodynamics are stable. Genetic counseling should be recommended and offered to all women and their partners and families prior to the planning of pregnancies. In AC patients, the numbers of obstetric adverse events during pregnancy, labor, and postpartum were comparable with those in age-matched women without a history of pregnancy. Vaginal delivery with epidural anesthesia and cardiac monitoring is reasonable and was preferred in most cases. Beta-blocker treatment resulted in low birth weights for gestational age. In women with a strong indication, the benefit of treatment with beta-blockers clearly outweighed the risk. Pregnancy-related maternal mortality and complications (VA, syncope, HF) were uncommon in AC patients. The degree of RV enlargement or dysfunction over time was not different between women who gave birth at least once and nulliparous women as controls. The number of completed pregnancies was not associated with an acceleration of AC pathology or an increased risk of VA or HF during follow-up. The overwhelming majority of infants were born alive without obstetric complications and stayed healthy during the reported follow-up periods.

In summary, there is no evidence to suggest that pregnancy should be discouraged in women with AC or that carrying an ICD should be regarded as a contraindication for pregnancy in AC.

## Supplementary Information

Supplementary Information: *Diagnostic criteria and differential diagnosis of AC*

## Abbreviations

AC: Arrhythmogenic cardiomyopathy
AVB: Atrioventricular block
CMR: Cardiovascular magnetic resonance
CVD: Cardiovascular disease
ECG: Electrocardiography
HF: Heart failure
ICD: Implantable cardioverter defibrillator
LV: Left ventricle (ventricular)
LVEF: Left ventricular ejection fraction
PPCM: Peripartal cardiomyopathy
PVC: Premature ventricular contraction
RV: Right ventricle (ventricular)
SCD: Sudden cardiac death
SVT: Supraventricular tachycardia
VA: Ventricular arrhythmia
VF: Ventricular fibrillation
VT: Ventricular tachycardia
VUS: Variant of unknown significance

## Declarations

For this article no studies with human participants or animals were performed by any of the authors. All studies performed were in accordance with the ethical standards indicated in each case.
