# Supplementary material for: Pregnancy in arrhythmogenic cardiomyopathy
Source: Herzschrittmacherther Elektrophysiol. 2021 May 25;32(2):186–98. doi: 10.1007/s00399-021-00770-7 (PMC8166670; doi:10.1007/s00399-021-00770-7)
Supplement: Supplementary file 1 — Supplementary Information: Diagnostic criteria and differential diagnosis of AC [file 399_2021_770_MOESM1_ESM.docx]

**Electronic supplementary material**

**Diagnostic Criteria of AC**

Since AC is not defined by a single diagnostic modality or clinical finding (*„gold standard“*), the correct diagnosis of AC relies on a catalogue of different criteria. In 1994, initial diagnostic criteria of AC were proposed by the International Task Force on Arrhythmogenic Right Ventricular Cardiomyopathy [43]. In 2010, these criteria were updated and modified by the same Collaborative Consortium [38], with the aim to improve the diagnostic sensitivity without a loss in specificity by introducing quantitative reference values for ECG, cardiac imaging and endomyocardial biopsy.

The catalogue of diagnostic criteria encompasses six categories of major or minor criteria for the diagnosis of AC:

1. ECG abnormalities of depolarization
2. ECG abnormalities of repolarization
3. Arrhythmia
4. Morphological and functional imaging
5. Structural abnormalities
6. Molecular and clinical genetics

A *„definite“* diagnosis of AC can be made when at least 2 major criteria, 1 major and 2 minor criteria, or 4 minor criteria from different diagnostic categories are present. One major and 1 minor criterion qualify for a *„borderline“* diagnosis of AC, whereas AC is considered *„possible“* in the presence of 1 major or 1 minor criterion [38, 64].

The diagnostic criteria modified in 2010 resulted in an improvement of diagnostic accuracy in AC and have been validated in prospective clinical studies and registries [8, 18, 20]. This culminated in further updates of diagnostic criteria that were proposed most recently in 2020 by International Expert Panels [17, 24, 60], with the aim to include new clinical and research knowledge and at the same time to simplify the decision process by eliminating criteria from the catalogue that no longer provided additional diagnostic value.

**Limitations**

There are several limitations to this review on pregnancy in AC. First, it retrospectively summarizes observational data from a limited number of patients and publications spanning over a period of 15 years and ranging from 2 to 120 women with 2 to 261 pregnancies with the majority being reported within the past 2 years [13, 27, 36, 48, 69]. Thus, data collection was heterogeneous and incomplete for several items. Apart from the variation in the number of patients and pregnancies included and the durations of follow-up, there was a broad spectrum of disease manifestation and severity before, during and after pregnancy, thus making it difficult to compare the outcome data in these heterogeneous populations.

In addition, several confounding factors play an important role. Women with severe manifestations of AC and higher risk of VA and/or HF at young age may have been advised and decided against pregnancy. Such discouragement of pregnancies would have resulted in an underrepresentation of high-risk female cohorts with AC in studies and registries dealing with the problem. This might have created a selection bias in favour of pregnancies in women with lower risk manifestations of AC. This would explain why (opposite to the expectations) nulliparous women with AC frequently appeared to be more severely afflicted when compared to those who gave birth [13, 48]. Further confounding factors may be geographical or ethnical heterogeneity with diverse genetic variants or environmental modifying factors, and differences in the diagnosis and treatment of AC.
